# Evaluation of Month of Birth in Neuromyelitis Optica Spectrum Disorders (NMSOD) and Multiple Sclerosis (MS)

**DOI:** 10.1155/2021/8874999

**Published:** 2021-06-10

**Authors:** Omid Mirmosayyeb, Mahdi Barzegar, Alireza Afshari-Safavi, Nasim Nehzat, Afshin Heidari, Parisa Emami, Vahid Shaygannejad

**Affiliations:** ^1^Isfahan Neurosciences Research Center, Isfahan University of Medical Sciences, Isfahan, Iran; ^2^Department of Neurology, School of Medicine, Isfahan University of Medical Sciences, Isfahan, Iran; ^3^Universal Council of Epidemiology (UCE), Universal Scientific Education and Research Network (USERN), Tehran University of Medical Sciences, Tehran, Iran; ^4^Department of Biostatistics and Epidemiology, Faculty of Health, North Khorasan University of Medical Sciences, Bojnurd, Iran

## Abstract

**Introduction:**

Multiple sclerosis (MS) and neuromyelitis optica spectrum disorder (NMO) are chronic immune-mediated diseases in the central nervous system (CNS). Environmental factors such as month of birth can be a trigger for these diseases. Therefore, we conducted this study to compare the months of birth in MS and NMOSD patients with the control group.

**Methods:**

In this cross-sectional study, 2345 patients with MS, 220 NMOSD patients, and 2174 healthy subjects were enrolled. Demographic information such as age, sex, month of birth, and education in three groups was extracted from the database. The associations between month of birth and MS were studied by binary logistic regression with adjusting for the year of birth.

**Results:**

There was a reduced birth rate in September-October in NMOSD (OR = 0.309, 95% CI: 0.150-0.636; *p* < 0.001) and MS patients (OR = 0.470, 95% CI: 0.374-0.591; *p* < 0.001) compared to the general population. The birth rate in March-April in MS was higher than the control group (OR = 1.613, 95% CI: 1.324-1.964; *p* < 0.001). There was no difference in the birth month distribution between the NMOSD and MS patients. No significant difference in MOB among different MS types was found.

**Conclusion:**

Our findings showed a decreasing risk of NMOSD and MS in individuals born in the autumn months and an increasing MS risk in spring. More studies are required to elucidate the association between the month of birth and risk of MS and NMOSD and the seasonality factors.

## 1. Introduction

Multiple sclerosis (MS) and neuromyelitis optica spectrum disorders (NMOSD) are chronic inflammatory diseases that involve the central nervous system (CNS) [1]. For many years, NMOSD was considered a variant of MS; however, discovering a disease-specific serum NMO-immunoglobulin G (IgG) antibody helped distinguish between these diseases [2]. More studies on clinical course, immunological features, and imaging findings provide clear evidence on differences between NMOSD and MS. Today, NMOS and MS are known as two distinct diseases [3–5].

The exact etiology of NMOSD and MS is still unclear. The interaction between genetic and environmental factors has been implicated as the probable underlying MS etiologies [6]. Environmental factors such as childhood residence, migration in early adolescence, and the distance of the place from the equator have an essential role in developing MS [7–9]. The last two decades have seen a growing trend towards the month of birth's (MoB) effect on the risk of autoimmune diseases [10–12]. However, the debate continues about the influence of MoB on the risk of MS in adulthood. Some studies showed an association between spring births and MS susceptibility [12–14]. On the other hand, there is some evidence on increasing MS risk in individuals born in the winter months and a protective effect when born in the autumn [15–17]. Some investigation results showed no difference in the month of birth pattern between MS and the general population [18, 19]. Moreover, some studies were confounded by some variables such as years and place of birth [20, 21].

Until now, far too little attention has been paid to the distribution of birth months within the NMOSD population. NMOSD has a strong association with other autoimmune disorders and shares similar triggers for the immune-mediated process. It can be hypothesized an association between MoB and NMOSD. Therefore, we set out this study to determine birth months' distribution in Iranian patients with NMOSD and MS and compare with the general population.

## 2. Methods

Data on the month and year of birth of 2565 patients with MS and NMOSD were extracted from the Isfahan Hakim MS database [22]. All patients were visited in the MS clinic of Kashani hospital, affiliated to Isfahan University of Medical Sciences, Isfahan, Iran. Diagnosis of MS and NMOSD was made by a neurologist with subspecialty training in MS (VS), based on McDonald criteria [23–25] or International consensus NMOSD diagnostic criteria [26]. Demographic and clinical information, including age, sex, marital status, education levels, number of relapses within the previous year, disease severity, and MS course, were extracted. The severity of diseases (both NMOSD and MS) was measured using the Expanded Disability Status Scale (EDSS) score [27].

The control data were extracted from two main healthcare networks of Isfahan city (number 1 and 2) attending the Isfahan University of Medical Sciences. Five centers affiliated at the healthcare network 1 and the latter five affiliated at the healthcare network 2 randomly were selected from the different urban areas using Random Allocation software. Therefore, each of the centers was provided with a particular code. Eventually, the information of 2174 healthy people was retrieved through a random selection of the participant based on their family code. Demographic data, including age, sex, marital status, education levels, medical history, month, and year of birth, were extracted. The exclusion criteria for both groups were diagnosis of autoimmune or neurological diseases rather than MS and NMOSD. This study was approved by the Ethics Committee of Isfahan University of Medical Sciences (Ethics code number IR.MUI.MED.REC.1398.403).

The obtained data were entered into the Statistical Package for Social Sciences (SPSS) version 25. The descriptive data were presented in mean, standard deviation, absolute numbers, and percentages. The chi-square test and independent *t*-test (or Mann–Whitney) were utilized for comparison between groups. The associations between month of birth and MS were studied by binary logistic regression with adjusting for the year of birth. Significance level after correcting with Bonferroni was *p* < 0.05/12 = 0.0042.

## 3. Results

The information of 5092 participants, including 220 NMOSD cases, 2345 MS, and 2174 healthy subjects, were evaluated.  Table 1 shows demographic and clinical features of individuals. The studied groups were statistically different in terms of marital status (*p* < 0.001) and educational level (*p* < 0.001). There were significant differences between various MS courses in EDSS score (*p* < 0.001) and the number of relapses in last year (*p* = 0.02).

**Figure 1:**
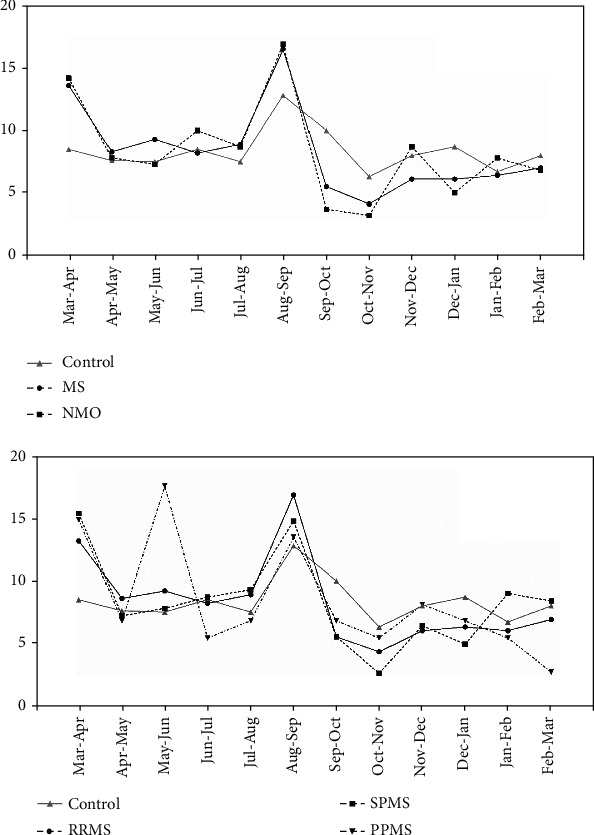
The percentage of birth in different months.

Figure 1 shows birth months' distribution in study groups. After adjusting for the year of birth, a reduced birth rate in September-October in NMOSD (OR = 0.309, 95% CI: 0.150-0.636; *p* < 0.001) and MS (OR = 0.470, 95% CI: 0.374-0.591; *p* < 0.001) compared to the general population was detected. The birth rate in March-April in MS was higher than the control group (OR = 1.613, 95% CI: 1.324-1.964; *p* < 0.001). There was no difference in the birth month distribution between the NMOSD and MS patients (Table 2).

Table 3 compares frequency and odds ratios by the MoB among different MS types and the general population. Significant increase of birth rate of RRMS patients in March-April (OR = 1.572, 95% CI: 1.282-1.927; *p* < 0.001) and August-September (OR = 1.314, 95% CI: 1.103-1.567; *p* < 0.001) and a reduction in September-October (OR = 0.482, 95% CI: 0.378-0.614; *p* = 0.002) compared to the reference population were found. SPMS birth rate was higher in March-April (OR = 1.773, 95% CI: 1.260-2.493; *p* = 0.001) and lower in September-October (OR = 0.410, 95% CI: 0.252-0.669; *p* < 0.001). We observed a significant increase in the birth rate in May-June (OR = 2.611, 95% CI: 1.391-4.903; *p* = 0.003) in PPMS compared to the control group. There was no significant difference in MOB among different MS types.

## 4. Discussion

In this study of the Iranian population, we found some evidence for variation in monthly birth rates in NMOSD and MS patients. Compared to control, there was a significantly increased risk of developing MS in the months of March-April. At the same time, a decreased risk for occurring NMOSD and MS in September-October was observed. These findings suggested the impact of month of birth on the development of NMOSD and MS in Iran.

Our MS patients' birth rate increased in spring and summer and dropped sharply in September-October, remaining steady during autumn and winter. This finding accords with the recent systematic review and meta-analysis, which showed that MS births in spring are higher than in autumn [28]. A large population-based study from Canada, Great Britain, Denmark, and Sweden showed a significant increase in MS birth in May and decreased in November [29]. Previous studies from Italy [30], Scotland [31], and Finland [32] also found that the birth in spring substantially increased the risk of future MS development.

The number of birth in all MS courses reached peaks during spring. Our findings showed a different pattern of birth-month among various MS courses compared to the general population. However, we were unable to find the month of birth influence on MS phenotype. In contrast to our finding, Sadovnick and colleagues found differences in May/November birth ratios between PPMS and RRMS. This inconsistency may be due to the small number of PPMS patients in our study that limited the power of our analysis.

We next investigated the birth-month patterns in NMOSD patients. To the best of our knowledge, the birth-monthly and seasonal pattern in NMOSD patients has not been evaluated so far. The number of birth in NMOSD patients reached peaks during spring and summer and decreased in autumn.

The relation between month of birth and autoimmune diseases may partly be explained by the fetal origin of the adult disease hypothesis. According to this, exposure to seasonal factors in the perinatal period can affect embryonic or fetal tissue structures and develop physical and psychological diseases [33]. Low exposure to UVB radiation in autumn and winter can lead to vitamin D deficiency. In this period, maternal insufficient vitamin D negatively influences the fetus' immunological and brain development. It increases the risk of lifetime MS in those born in spring and summer [34–37]. A study on cord blood showed that infants born in May had greater CD4+ and CD8+ and lower 25-hydroxyvitamin D levels than those born in November [38].

In contrast to MS, there is much less information about the effect of low vitamin D on the susceptibility to NMOSD. A lower 25 (OH) D_1-3_ levels in NMOSD patients than the healthy controls are demonstrated [39–41]. Moreover, an inverse relationship between vitamin D level and disease activity has been suggested [39, 40]. Experimental and in vitro studies have shown that vitamin D is involved in regulating both innate and acquired immunity in NMOSD [42, 43]. However, no study has evaluated the impact of inadequate vitamin D during pregnancy on the risk of NMOSD in adulthood.

Latitude and climate features are other possible factors that contribute to the monthly distribution of autoimmune diseases. Our data are consistent with those conducted in other countries in the northern hemisphere, where an increase in MS birth in spring and summer was observed [30, 44]. Both genetic and environmental factors are responsible for explaining the possible effect of latitude on autoimmunity. A systematic review found latitudinal gradient only in European-descent regions suggesting related genetic factors [45]. On the other hand, changing the likelihood of developing MS following immigration after birth shows the role of associated environmental factors. Although there is a latitude gradient for the prevalence of MS [45], no clear association between latitude and NMOSD has been found [46]. Further studies are needed to declare the relationship between latitude and NMOSD and its effect on these patients' MoB pattern.

The pattern of MS-birth in regions with an extreme climate, similar to Isfahan, showed a substantial difference between summer and winter [47]. However, studies in countries with milder climates found no relation between MS and seasonality [15, 16]. Climate and latitude mediated this association through vitamin D and nonvitamin pathways. Individuals in regions with high latitude or cold desert climate received insufficient UVB light from autumn to winters to make vitamin D3 in their skin during these months.

Our study has some limitations. The control group was not matched with patients. However, it seems that there was no significant difference between study groups in age and sex. Our patients were enrolled from one MS center, which limited our results to other MS patients. One of the strengths of this study is adjusting for age of birth as an essential confounder [20].

In conclusion, this study provided additional support for an association between month of birth and MS development. Our findings suggest a possible association between month of birth and risk of NMOSD; however, the evidence is still insufficient to draw a certain conclusion. Because of differences between NMOSD and MS in environmental and genetic risk factors [46, 48], it is impossible to apply the suggested seasonal factors to the NMOSD population. Further studies are recommended to elucidate the relation between month of birth and disease development of NMOSD and its seasonal factors.

## Figures and Tables

**Table 1:** Demographic and clinical features of participants.

Variables	NMOSD (*n* = 220)	MS (*n* = 2345)			Control group (*n* = 2174)	*p* value
		RRMS (*n* = 1918)	PPMS (*n* = 74)	SPMS (*n* = 353)		
Age	36.65 ± 10.62	37.85 ± 9.92	45.82 ± 10.81	44.98 ± 9.28	35.92 ± 24.44	0.17
Gender (M/F)	24.1/75.9	18.2/81.8	45.9/54.1	24.1/75.9	29.1/78.1	0.12
Marital status (married/single)	66.2/28.8	73.3/23.3	79.7/14.9	78.6/14.5	67.2/32.8	<0.001
Education (basic/advanced)	128/92	957/961	56/18	263/90	1754/390	<0.001
EDSS	1.85 ± 1.88	1.28 ± 0.9	4.55 ± 2.42	5.46 ± 2.42	—	<0.001
Relapses within a previous year	0.62 ± 0.47	0.68 ± 0.34	0.53 ± 0.31	0.62 ± 0.33	—	0.02

M: male; F: female; NMOSD: neuromyelitis optica spectrum disorders; RRMS: relapsing-remitting multiple sclerosis; PPMS: primary progressive multiple sclerosis; SPMS: secondary progressive multiple sclerosis; EDSS: Expanded Disability Status Scale. *p* value of less than 0.05 was considered as a significant level.

**Table 2:** Comparison of numbers of monthly births among study groups.

Month	MS vs. control		NMOSD vs. control		MS vs. NMOSD	
	OR (95% CI)	*p*	OR (95% CI)	*p*	OR (95% CI)	*p*
March-April	1.613 (1.324-1.964)	<0.001	1.712 (1.136-2.582)	0.010	1.062 (0.713-1.581)	0.769
April-May	1.162 (0.924-1.460)	0.199	1.082 (0.641-1.828)	0.767	0.932 (0.556-1.562)	0.789
May-June	1.228 (0.986-1.530)	0.066	0.958 (0.561-1.637)	0.876	0.780 (0.460-1.323)	0.356
June-July	0.909 (0.732-1.129)	0.389	1.154 (0.723-1.841)	0.549	1.269 (0.797-2.020)	0.315
July-August	1.253 (1.00-1.571)	0.050	1.228 (0.744-2.026)	0.423	0.979 (0.599-1.602)	0.934
August-September	1.266 (1.067-1.502)	0.007	1.318 (0.905-1.921)	0.150	1.041 (0.719-1.507)	0.831
September-October	0.470 (0.374-0.591)	<0.001	0.309 (0.150-0.636)	0.001	0.658 (0.317-1.363)	0.260
October-November	0.694 (0.522-0.921)	0.012	0.534 (0.246-1.163)	0.114	0.770 (0.353-1.681)	0.512
November-December	0.812 (0.637-1.035)	0.093	1.181 (0.716-1.948)	0.516	1.453 (0.881-2.397)	0.143
December-January	0.731 (0.576-0.927)	0.010	0.592 (0.316-1.109)	0.101	0.809 (0.431-1.519)	0.510
January-February	1.045 (0.812-1.345)	0.734	1.285 (0.757-2.180)	0.353	1.230 (0.729-2.073)	0.438
February-March	0.872 (0.692-1.098)	0.245	0.857 (0.495-1.485)	0.582	0.983 (0.568-1.700)	0.950

**(a):** 

Month	RRMS vs. control		SPMS vs. control		PPMS vs. control	
	OR (95% CI)	*p*	OR (95% CI)	*p*	OR (95% CI)	*p*
March-April	1.572 (1.282-1.927)	<0.001	1.773 (1.260-2.493)	0.001	1.741 (0.895-3.385)	0.102
April-May	1.185 (0.937-1.497)	0.156	1.041 (0.660-1.641)	0.862	0.989 (0.390-2.509)	0.981
May-June	1.232 (0.981-1.547)	0.073	1.018 (0.657-1.577)	0.936	2.611 (1.391-4.903)	0.003
June-July	0.920 (0.734-1.153)	0.470	0.927 (0.612-1.402)	0.718	0.566 (0.203-1.576)	0.276
July-August	1.245 (0.986-1.571)	0.065	1.379 (0.909-2.93)	0.131	1.002 (0.395-2.541)	0.997
August-September	1.314 (1.103-1.567)	0.002	1.023 (0.734-1.424)	0.895	0.937 (0.473-1.856)	0.853
September-October	0.482 (0.378-0.614)	<0.001	0.410 (0.252-0.669)	<0.001	0.513 (0.204-1.292)	0.157
October-November	0.714 (0.534-0.954)	0.023	0.454 (0.225-0.914)	0.027	0.991 (0.352-2.786)	0.986
November-December	0.793 (0.615-1.022)	0.074	0.914 (0.566-1.476)	0.714	1.214 (0.513-2.870)	0.659
December-January	0.747 (0.585-0.955)	0.020	0.614 (0.363-1.038)	0.069	0.879 (0.347-2.226)	0.785
January-February	0.962 (0.738-1.255)	0.777	1.656 (1.074-2.552)	0.022	0.981 (0.349-2.757)	0.972
February-March	0.861 (0.676-1.095)	0.223	1.068 (0.697-1.635)	0.764	0.331 (0.080-1.366)	0.126

**(b):** 

Month	RRMS vs. SPMS		RRMS vs. PPMS		SPMS vs. PPMS	
	OR (95% CI)	*p*	OR (95% CI)	*p*	OR (95% CI)	*p*
March-April	1.134 (0.818-1.572)	0.450	1.114 (0.577-2.150)	0.747	0.982 (0.486-1.986)	0.961
April-May	0.879 (0.564-1.369)	0.568	0.835 (0.331-2.106)	0.703	0.950 (0.351-2.569)	0.920
May-June	0.819 (0.534-1.256)	0.360	2.099 (1.126-3.913)	0.020	2.563 (1.253-5.245)	0.010
June-July	1.00 (0.662-1.513)	0.998	0.611 (0.220-1.700)	0.345	0.611 (0.208-1.789)	0.369
July-August	1.113 (0.744-1.665)	0.602	0.809 (0.321-2.038)	0.652	0.726 (0.273-1.931)	0.522
August-September	0.785 (0.567-1.085)	0.143	0.720 (0.365-1.421)	0.343	0.917 (0.442-1.903)	0.817
September-October	0.845 (0.509-1.403)	0.516	1.057 (0.416-2.685)	0.908	1.250 (0.451-3.466)	0.668
October-November	0.638 (0.316-1.288)	0.210	1.393 (0.495-3.922)	0.530	2.184 (0.654-7.292)	0.204
November-December	1.145 (0.711-1.844)	0.578	1.520 (0.643-3.588)	0.340	1.327 (0.518-3.398)	0.555
December-January	0.822 (0.486-1.390)	0.464	1.176 (0.464-2.979)	0.732	1.432 (0.511-4.013)	0.495
January-February	1.739 (1.140-2.652)	0.010	1.030 (0.368-2.883)	0.955	0.593 (0.203-1.734)	0.339
February-March	1.235 (0.808-1.889)	0.329	0.382 (0.093-1.579)	0.184	0.310 (0.072-1.327)	0.114

## Data Availability

The data is available but is not included in the paper.
